# Four Molybdenum-Dependent Steroid C-25 Hydroxylases: Heterologous Overproduction, Role in Steroid Degradation, and Application for 25-Hydroxyvitamin D_3_ Synthesis

**DOI:** 10.1128/mBio.00694-18

**Published:** 2018-06-19

**Authors:** Christian Jacoby, Jens Eipper, Markus Warnke, Oliver Tiedt, Mario Mergelsberg, Hans-Joachim Stärk, Birgit Daus, Zaira Martín-Moldes, María Teresa Zamarro, Eduardo Díaz, Matthias Boll

**Affiliations:** a Faculty of Biology–Microbiology, Albert-Ludwigs-Universität Freiburg, Freiburg, Germany; b Department of Analytical Chemistry, Helmholtz Centre for Environmental Research UFZ, Leipzig, Germany; c Department of Microbial and Plant Biotechnology, Centro de Investigaciones Biológicas, CSIC, Madrid, Spain; University of California, Irvine

**Keywords:** alkyl hydroxylases, anaerobic catabolic pathways, molybdenum enzymes, sterols, vitamin D3 biosynthesis

## Abstract

Side chain-containing steroids are ubiquitous constituents of biological membranes that are persistent to biodegradation. Aerobic, steroid-degrading bacteria employ oxygenases for isoprenoid side chain and tetracyclic steran ring cleavage. In contrast, a Mo-containing steroid C-25 dehydrogenase (S25DH) of the dimethyl sulfoxide (DMSO) reductase family catalyzes the oxygen-independent hydroxylation of tertiary C-25 in the anaerobic, cholesterol-degrading bacterium Sterolibacterium denitrificans. Its genome contains eight paralogous genes encoding active site α-subunits of putative S25DH-like proteins. The difficult enrichment of labile, oxygen-sensitive S25DH from the wild-type bacteria and the inability of its active heterologous production have largely hampered the study of S25DH-like gene products. Here we established a heterologous expression platform for the three structural genes of S25DH subunits together with an essential chaperone in the denitrifying betaproteobacterium Thauera aromatica K172. Using this system, S25DH_1_ and three isoenzymes (S25DH_2_, S25DH_3_, and S25DH_4_) were overproduced in a soluble, active form allowing a straightforward purification of nontagged αβγ complexes. All S25DHs contained molybdenum, four [4Fe-4S] clusters, one [3Fe-4S] cluster, and heme B and catalyzed the specific, water-dependent C-25 hydroxylations of various 4-en-3-one forms of phytosterols and zoosterols. Crude extracts from T. aromatica expressing genes encoding S25DH_1_ catalyzed the hydroxylation of vitamin D_3_ (VD_3_) to the clinically relevant 25-OH-VD_3_ with >95% yield at a rate 6.5-fold higher than that of wild-type bacterial extracts; the specific activity of recombinant S25DH_1_ was twofold higher than that of wild-type enzyme. These results demonstrate the potential application of the established expression platform for 25-OH-VD_3_ synthesis and pave the way for the characterization of previously genetically inaccessible S25DH-like Mo enzymes of the DMSO reductase family.

## IMPORTANCE

Steroids are ubiquitous bioactive compounds, some of which are considered an emerging class of micropollutants. Their degradation by microorganisms is the major process of steroid elimination from the environment. While oxygenase-dependent steroid degradation in aerobes has been studied for more than 40 years, initial insights into the anoxic steroid degradation have only recently been obtained. Molybdenum-dependent steroid C-25 dehydrogenases (S25DHs) have been proposed to catalyze oxygen-independent side chain hydroxylations of globally abundant zoo-, phyto-, and mycosterols; however, so far, their lability has allowed only the initial characterization of a single S25DH. Here we report on a heterologous gene expression platform that allowed for easy isolation and characterization of four highly active S25DH isoenzymes. The results obtained demonstrate the key role of S25DHs during anoxic degradation of various steroids. Moreover, the platform is valuable for the efficient enzymatic hydroxylation of vitamin D_3_ to its clinically relevant C-25-OH form.

## INTRODUCTION

Steroids are a ubiquitously occurring class of highly hydrophobic compounds that in eukaryotes act as hormones and essential components of biological membranes (1, 2). Due to their wide abundance and biological activity, the elimination of steroids from the environment is of global relevance (3, 4). Biodegradation of steroids is hampered by their low water solubility and by the complex tetracyclic core structure comprising quaternary carbon atoms. Only microorganisms are capable of fully degrading steroids to CO_2_. In the presence of oxygen, degradation of steroids heavily depends on oxygenase-dependent hydroxylation and ring cleavage reactions (5, 6).

**FIG 1:**
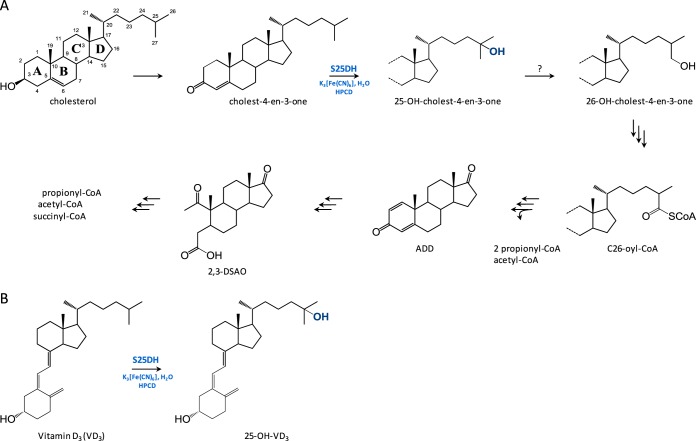
(A) Proposed anoxic degradation pathway of cholesterol in Sterolibacterium denitrificans. The activation of the side chain is mediated by S25DH, which hydroxylates the tertiary C-25 carbon with water. ADD, androsta-1,4-diene-3-one; 2,3-DSAO, 1,17-dioxo-2,3-*seco*-androstan-3-oic acid. (B) Conversion of vitamin D_3_ (VD_3_) to 25-OH-VD_3_ by S25DH using ferricyanide as the electron acceptor.

Anaerobic steroid degradation has been studied in only a few denitrifying proteobacteria with the cholesterol-degrading *Sterolibacterium denitrificans* serving as a model organism (7). Recent studies revealed a patchwork pathway for anaerobic steroid degradation (8, 9). As in aerobic cholesterol-degrading organisms, cholest-4-en-3-one is formed as the first intermediate from cholesterol in S. denitrificans (Fig. 1A) (10). The subsequent hydroxylation of the side chain with water that occurs at tertiary C-25 is then catalyzed by molybdenum (Mo)-dependent steroid C-25 dehydrogenase (S25DH) (10, 11), and not at primary C-26 as observed in the oxygenase-dependent pathway. The next step involves a formal shift of the hydroxyl group from the tertiary C-25 to primary C-26 by an unknown enzyme (8, 12). Further degradation to androsta-1,4-diene-3,17-dione (ADD) proceeds via oxidation and activation to a C-26-oyl-coenzyme A (CoA) component, followed by modified β-oxidation like reaction sequences (Fig. 1) (8, 13). Finally, cleavage of the steran rings A and B proceeds in the so-called 2,3-*seco*-pathway involving ring-cleaving hydrolases (14, 15); degradation of rings C and D appears to be similar in aerobic and anaerobic steroid-degrading bacteria, again using hydrolytic enzymes (Fig. 1) (16).

A S25DH was initially isolated and characterized by Dermer and Fuchs (11) as a molybdenum cofactor (MoCo)-containing enzyme of the dimethyl sulfoxide reductase (DMSOR) family of Mo enzymes. The enzyme has an αβγ architecture comprising a 108-kDa (α), 38-kDa (β), and 23-kDa (γ) subunit. The catalytically active α-subunit contains a molybdo-*bis*-pyranopterin guanine dinucleotide (Mo-*bis*PGD) cofactor and a [4Fe-4S] cluster; the β-subunit harbors four additional FeS clusters, and the γ-subunit contains a b-type heme. Like other members of the DMSOR family, S25DH is proposed to be located in the periplasm. It was proposed that a chaperone (SdhD) is involved in proper folding and probably in MoCo insertion (11). S25DH together with ethylbenzene dehydrogenase (EbDH) (17, 18) and *p*-cymene dehydrogenase (19) forms a phylogenetic subcluster within class II of the DMSOR family, that hydroxylate alkyl side chains of steroids or aromatic compounds with water (see Fig. S1 in the supplemental material) (20). Attempts for heterologous/homologous production of S25DH/EbDH subclass members have failed so far (21), which prevented an easy enrichment procedure, as well as the generation of molecular variants.

10.1128/mBio.00694-18.1 FIG S1  Phylogenetic tree of active site α-subunits from S25DH-like enzymes inside the DMSOR family of MoCo-containing enzymes. Download FIG S1, PDF file, 0.5 MB. Copyright © 2018 Jacoby et al. 2018 Jacoby et al. This content is distributed under the terms of the Creative Commons Attribution 4.0 International license.

A recent study revealed that S25DH is capable of hydroxylating vitamin D_3_ (VD_3_) to the clinically relevant 25-OH-VD_3_ (Fig. 1B) (22). This activity was dependent on 2-hydroxypropyl-β-cyclodextrin that is known to promote the isomerization to pre-VD_3_ (23), the assumed actual substrate of S25DH. In contrast to cytochrome P450-dependent hydroxylation of VD_3_, S25DH-dependent catalysis is independent of an electron donor system and requires only the electrochemically regenerative K_3_[Fe(CN)_6_] (ferricyanide) as an electron acceptor (22). Though S25DH serves as a promising catalyst for the synthesis of 25-OH-VD_3_, there is a high demand for heterologous expression and for improving the enrichment of S25DH-like enzymes with regard to activities and stabilities.

**FIG 2:**
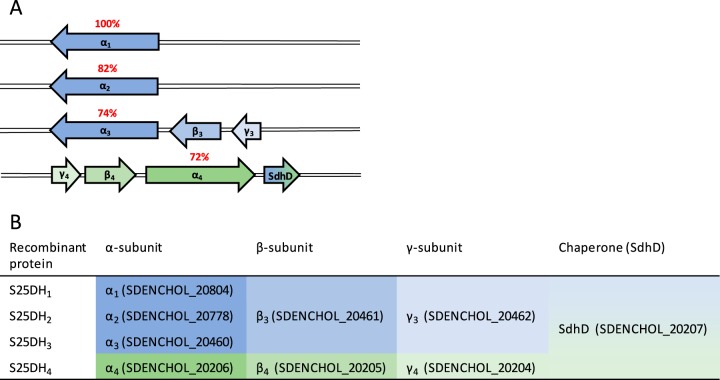
Genes encoding S25DH subunits and chaperone. (A) Arrangement of genes encoding putative α_1-4_ (MoCo-containing), β_3_,_4_ (4Fe-4S cluster-containing), and γ_3,4_ (heme b-containing) subunits, as well as the chaperone (SdhD) of S25DHs. (B) Constructs prepared for the heterologous production of S25DH_1_, S25DH_2_, S25DH_3_, and S25DH_4_.

*Sterolibacterium* denitrificans is capable of degrading phyto- and mycosterols such as β-sitosterol, stigmasterol, or ergosterol with modifications in the isoprenoid side chain (for structures, see Table 1), but the only cholest-4-en-3-one-converting S25DH studied so far is unable to convert any of the 4-en-3-one analogues of these growth substrates (8, 11). In addition to the gene encoding the active site α-subunit of this S25DH (henceforth referred to as α_1_ subunit of S25DH_1_, gene accession number SDENCHOL_20805), the genome contains seven paralogous genes encoding putative S25DH-like enzymes, all affiliating with the class II DMSOR family (α_2_-α_8_) (8, 11). In particular, the predicted active site α_2–4_ (amino sequence identities to α_1 _of 72 to 82%) have been hypothesized to represent the active site subunits of S25DH_2_, S25DH_3_, and S25DH_4_ involved in C-25 hydroxylation of steroids with modified isoprenoid side chains (Fig. 2A). This assumption is based on their differential abundance during growth on different steroids such as β-sitosterol or ergosterol (8); the role of the other four putative S25DHs (S25DH_5_, S25DH_6_, S25DH_7_, and S25DH_8_) is unclear (8). Notably, there are fewer genes encoding the βγ-subunit components than for the α-subunits in the genome of S. denitrificans (Fig. 2A), suggesting that S25DHs with different α-subunits share common βγ-subunit components. S25DH_1_ from S. denitrificans is composed of the α_1_β_3_γ_3_-subunits (Fig. 2A). Enriched S25DH_1_ always contained impurities of other α-subunits, which made a clear assignment of activities to individual α-subunits problematic (11, 22).

**TABLE 1:**
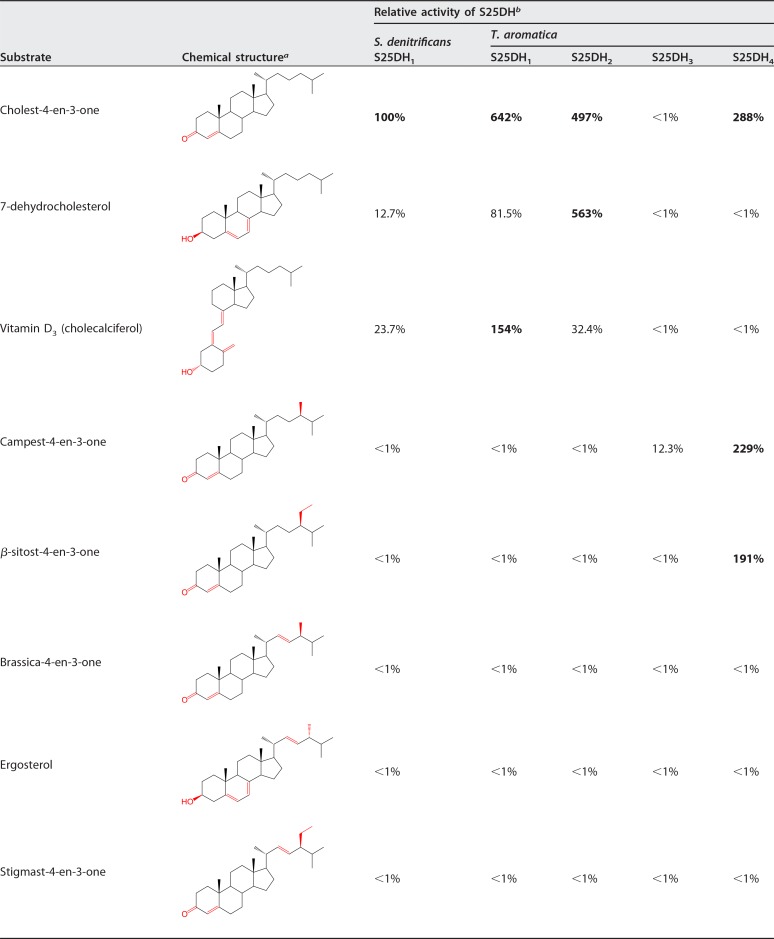
Relative activities of S25DH_1_ in cell extracts of Sterolibacterium denitrificans cells grown with cholesterol compared to those in cell extracts from T. aromatica producing S25DH_1_, S25DH_2_, S25DH_3_, and S25DH_4_

^*a*^Structural differences are shown in red.

^*b*^One hundred percent activity corresponds to 1.87 nmol min^−1^ mg^−1^ as observed for cholest-4-en-3-one conversion by extracts from wild-type bacteria grown with cholesterol. The boldface values indicate activities higher than 100%.

In this work, we aimed to elucidate the unknown function of Mo-containing S25DH isoenzymes in S. denitrificans in anaerobic steroid degradation and to explore their potential use for 25-OH-VD_3_ synthesis. For this purpose, an expression platform for S25DH and related enzymes was established in the denitrifying betaproteobacterium Thauera aromatica yielding S25DHs with high yields and specific activities. With this tool, four recombinant S25DHs were isolated and characterized; their applicability as catalysts for 25-OH-VD_3_ synthesis was probed.

## RESULTS

### Heterologous production of S25DH_1_.

The previously established five-step enrichment of the oxygen-sensitive S25DH_1_ (α_1_β_3_γ_3_ complex) from wild-type *Sterolibacterium* denitrificans grown with cholesterol always gave low yields, low specific activities, and a partially degraded α_1_-subunit (11, 22, 24). Moreover, the enriched enzyme frequently contained impurities from other S25DH α-subunits next to the α_1_-subunit, which did not allow unambiguous assignment of activities to individual gene products (11, 22). These findings motivated us to establish a platform for actively expressing the genes encoding S25DH_1_ and related enzymes. For this reason, the genes encoding the α_1_β_3_γ_3_-subunits, together with the putative chaperone (henceforth referred to as SdhD) (Fig. 2) were cloned into the broad-host-range plasmid pIZ1016. This construct contained the twin-arginine translocation (TAT) secretion sequence at the N terminus of the α_1_-subunit; to avoid any possible negative effect on αβγ complex formation, we avoided the use of a tagged subunit. Heterologous production of the resulting α_1_β_3_γ_3_SdhD construct was tested in Escherichia coli strains BL21 and Top 10, *Azoarcus* sp. strain CIB, and Thauera aromatica K172.

Heterologous production of the α_1_-subunit alone in the presence or absence of SdhD was not monitored in this work, as preliminary experiments indicated that such constructs did not result in the formation of soluble/active proteins. Expression of the α_1_β_3_γ_3_SdhD-encoding genes did not give soluble gene products in either of the two E. coli strains, and consequently, virtually no formation of 25-hydroxy-cholest-4-en-3-one was observed. However, after heterologous production of α_1_β_3_γ_3_SdhD in *Azoarcus* sp. CIB (25) and T. aromatica (26), anaerobically prepared cell extracts from both species showed the conversion of 0.5 mM cholest-4-en-3-one (0.5 mM) to 25-hydroxy-cholest-4-en-3-one; this conversion was dependent on time, protein, K_3_[Fe(CN)_6_] (5 mM), and 2-hydroxypropyl-β-cyclodextrin (HPCD) (9% wt/vol) and was observed only with constructs containing all four α_1_β_3_γ_3_SdhD components. The specific activities of recombinant S25DH_1_ were maximally 3.3 nmol min^−1^ mg^−1^ in cell extracts from *Azoarcus* sp. CIB and 12 nmol min^−1^ mg^−1^ in cell extracts from T. aromatica. Remarkably, both specific activities were 176% and more than 640% of the activity in cell extracts of cholesterol-grown S. denitrificans (1.87 nmol min^−1^ mg^−1^), respectively, demonstrating overproduction of S25DH_1_ in the *Azoarcus* and *Thauera* species (Table 1). Under anoxic conditions, loss of recombinant S25DH_1_ activity was less than 10% for 1 week at 4°C. In the presence of 5 mM K_3_[Fe(CN)_6_], activity in T. aromatica crude extracts was stable in air at 30°C for 5 to 8 h but decreased to 50% after 24 h.

**FIG 3:**
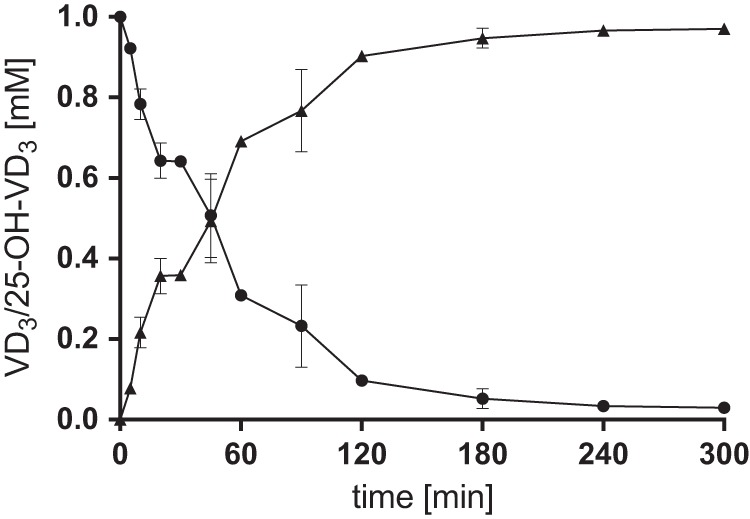
Aerobic conversion of 1 mM VD_3_ to 25-OH-VD_3_ by crude extracts of Thauera aromatica K172 producing S25DH_1_ (12 mg ml^−1^). Symbols: ● VD_3_; ▲, 25-OH-VD_3_.

T. aromatica crude extracts producing S25DH_1_ catalyzed the conversion of VD_3_ to 25-OH-VD_3_ at 2.8 nmol min^−1^ mg^−1^ that was 6.5-fold higher compared to wild-type extract (0.43 nmol min^−1^ mg^−1^). This increased VD_3_ conversion rate shortened the conversion of 1 mM VD_3_ correspondingly (90% conversion in 2 h by 12 mg ml^−1^ crude extracts) (Fig. 3), allowing 25-OH-VD_3_ synthesis even in air without loss of activity. The absence of 25-OH-VD_3_-converting enzymes in T. aromatica made the addition of AgNO_3_ for blocking follow-up enzymes of the cholesterol degradation pathway dispensable (22). Recombinant S25DH also converted 7-dehydrocholesterol to its 25-OH form, as had also been reported for the wild-type enzyme (11); no analogues with modifications in the isoprenoid side chain were converted (Table 1).

### Heterologous production of S25DH_2_, S25DH_3_, and S25DH_4_ isoenzymes in T. aromatica.

The results obtained indicated that T. aromatica K172 represents the most suitable host for the heterologous production of S25DH-like enzymes. For this reason, we used this strain for the production of three additional S25DH-like proteins that contain the α_2-4_ active site subunits, respectively. The constructs were cloned into pIZ1016 in a manner to give S25DH_2_, S25DH_3_, and S25DH_4_ with the compositions α_2_β_3_γ_3_, α_3_β_3_γ_3_, and α_4_β_4_γ_4_ referred to as S25DH_2_, S25DH_3_ and S25DH_4_ (Fig. 2). Notably, the β_3_γ_3_ and β_4_γ_4_ subunits are almost identical (99% sequence identity), and the coexpression of their genes with those encoding individual α-subunits was chosen to facilitate the cloning procedure. In all cases, SdhD was coproduced with the individual S25DH isoenzymes.

Using cell extracts from T. aromatica producing the individual S25DH-like gene products, we tested the conversion of cholest-4-en-3-one, 7-dehydrocholesterol, vitamin D_3_, campest-4-en-3-one, brassica-4-en-3-one, ergosterol, β-sitost-4-en-3-one, and stigmast-4-en-3-one as possible substrates. The time-dependent formation of products was analyzed by ultrahigh-performance liquid chromatography (UPLC) separation coupled to diode array detection; identification was by UPLC coelution with standards in addition to electrospray ionization quadrupole time of flight mass spectrometry (ESI-QTOF-MS) detection. For UPLC chromatograms of conversions, see Fig. S2 in the supplemental material; for relative specific activities, see Table 1.

10.1128/mBio.00694-18.2 FIG S2  UPLC chromatograms showing the conversion of various steroid substrates by different recombinant S25DHs from Sterolibacterium denitrificans heterologously produced in T. aromatica. Download FIG S2, PDF file, 0.2 MB. Copyright © 2018 Jacoby et al. 2018 Jacoby et al. This content is distributed under the terms of the Creative Commons Attribution 4.0 International license.

Using cell extracts of T. aromatica producing S25DH_2_, the rate of 25-OH-7-dehydrocholesterol formation was slightly higher than conversion of cholest-4-en-3-one. This extract converted VD_3_ to 25-OH-VD_3_ at a higher rate than S. denitrificans extracts grown with cholesterol, albeit the activity was only around 20% of recombinant S25DH_1_ (Table 1). Previous differential proteome analysis showed that the α_3_-subunit of an S25DH (SDENCHOL_20460) was most abundant during growth with ergosterol containing a Δ22 double bond in the side chain (8). Unexpectedly, T. aromatica expressing the S25DH_3_ formed only traces of 25-OH-ergosterol from ergosterol. Likewise, brassica-4-en-3-one, an ergosterol analogue with AB rings identical to those in cholest-4-en-3-one was virtually not converted. Surprisingly, campest-4-en-3-one, which lacks the double bond in the side chain but contains an additional methyl branch at (*R*)-configured C-24, was the only steroid substrate tested that was converted by S25DH_3_ to its 25-OH-form in significant amounts (Table 1). The α_4_-subunit was found upregulated in S. denitrificans cells grown with β-sitosterol, a cholesterol analogue with an additional ethyl branch at C-24 (8). In full agreement, T. aromatica cell extracts producing S25DH_4_ converted β-sitost-4-en-3-one and the structurally related campest-4-en-3-one, both with an (*R*)-configured tertiary C-24; cholest-4-en-3-one lacking an additional alkyl substituent at this position was also converted at a slightly higher rate (Table 1).

### Enrichment, activity, and composition of recombinant S25DH_2_, S25DH_3_, and S25DH_4_.

The results obtained so far indicated a higher heterologous production of S25DH_1_ and probably other S25DHs in *Azoarcus* sp. CIB and T. aromatica K172 than in wild-type S. denitrificans. As a result, only two out of five chromatographic enrichment steps described for purification from the wild-type bacteria were necessary. The two steps were DEAE anion-exchange chromatography and affinity chromatography on Reactive Red agarose (11, 22); we initially tested the purification of the recombinant S25DH_1_ from both strains.

With *Azoarcus* sp. CIB extracts, five protein bands were obtained after the two-step purification procedure (Fig. S3). The bands migrating at 110, 40, and 25 kDa were clearly assigned to the α_1_β_3_γ_3_-subunits, whereas those migrating between 50 and 55 kDa represent truncated α_1_ degradation products, as observed during purification from S. denitrificans (11, 22). The two degradation products were estimated to make more than 80% of the total amount of the α_1_-subunit. This finding suggests that expression in *Azoarcus* sp. CIB produced predominantly degraded S25DH_1_ (see Fig. S3 in the supplemental material).

10.1128/mBio.00694-18.3 FIG S3  SDS-PAGE (12.5%) of the active fractions obtained during enrichment of S25DH_1_ (α_1_β_3_γ_3_-subunits) in *Azoarcus* sp. strain CIB. Download FIG S3, PDF file, 0.2 MB. Copyright © 2018 Jacoby et al. 2018 Jacoby et al. This content is distributed under the terms of the Creative Commons Attribution 4.0 International license.

**FIG 4:**
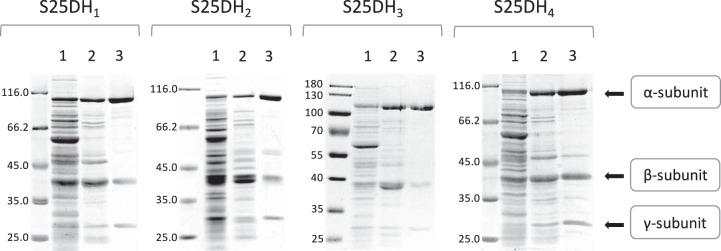
Enrichment of recombinant S25DHs. SDS-PAGE analysis of S25DH activity-containing fractions during the enrichment of recombinant S25DH_1_, S25DH_2_, S25DH_3_, and S25DH_4_ from T. aromatica cell extracts. Lane 1; 20 µg supernatant after centrifugation at 150,000 × *g*; lane 2, 10 µg protein after DEAE-Sepharose chromatography; lane 3, 5 µg protein obtained after Reactive Red chromatography. The positions of the corresponding α-, β- and γ-subunits of S25DHs are indicated by arrows to the right of the S25DH_4_ gel. The positions of molecular size markers (in kilodaltons) are indicated to the left of the gels.

Purification of S25DH_1_ from T. aromatica extracts revealed a highly enriched (purity >95%) α_1_β_3_γ_3_ complex with an almost perfect 1:1:1 ratio of the three subunits (Fig. 4). ESI-QTOF-MS analysis of tryptic digestion products of the three excised protein bands identified the expected α_1_β_3_γ_3_ subunits (see Table S1 in the supplemental material). Most importantly, when prepared from T. aromatica K172, α_1_-degradation products were negligible. These findings explain why the specific activity of recombinant S25DH_1_ in *Azoarcus* sp. CIB extracts was—though still higher than in S. denitrificans—significantly lower than in T. aromatica.

10.1128/mBio.00694-18.5 TABLE S1  Mass spectrometric analysis of S25DH_1_ from S. denitrificans heterologously produced in T. aromatica K172. Download TABLE S1, DOCX file, 0.01 MB. Copyright © 2018 Jacoby et al. 2018 Jacoby et al. This content is distributed under the terms of the Creative Commons Attribution 4.0 International license.

Based on the results obtained with S25DH_1_, the S25DH_2_, S25DH_3_, and S25DH_4_ isoenzymes were similarly purified after production of the individual enzymes in T. aromatica. During purification of the three S25DHs, the activity-containing fractions were assayed with the following substrates: cholest-4-en-3-one (S25DH_2_ and S25DH_4_) and campest-4-en-3-one (S25DH_3_). In all cases, predominantly nondegraded S25DH complexes were produced; only with S25DH_2_ a minor formation of α_2_-degradation products (<10%) was observed, as evidenced by a double band around 50 to 55 kDa (Fig. 4). The specific activities determined were highest with S25DH_1_/cholest-4-en-3-one (395 nmol min^−1^ mg^−1^); the enrichment factors varied between 15-fold (S25DH_4_) and 38-fold (S25DH_1_) with yields between 27 and 38% (Table 2).

**TABLE 2:** Enrichment of four S25DHs starting from 5 g (wet weight) recombinant T. aromatica

Enzyme and fraction	Protein (mg)	Sp act (nmol min^−1^ mg^−1^)^a^	Enrichment factor	Yield (%)
S25DH_1_				
Soluble fraction	645	11.2		100
DEAE + Reactive Red	6.6	395	35.3	36
S25DH_2_				
Soluble fraction	546	9.3		100
DEAE + Reactive Red	9	151	16.2	27
S25DH_3_				
Soluble fraction	590	0.21		100
DEAE + Reactive Red	10.2	4.6	21.9	38
S25DH_4_				
Soluble fraction	552	6.4		100
DEAE + Reactive Red	10	97	15.1	27

a Specific activities were determined by the conversion of cholest-4-en-3-one for S25DH_1_, S25DH_2_, and S25DH_4_ and of campest-4-en-3-one for S25DH_3_.

The subunit architecture and native molecular masses of the S25DHs were determined by size exclusion chromatography as follows: 167 ± 5 kDa for S25DH, S25DH_2_, and S25DH_3 _and 160 ± 5 kDa for S25DH_4_. These values clearly point to an αβγ composition of all four heterologously produced S25DHs.

### Metal content of recombinant S25DHs.

On the basis of the results of previous metal analyses with wild-type S25DH_1_ enzyme and on the conserved binding motifs of the individual cofactors, the purified recombinant S25DH_1_, S25DH_2_, S25DH_3_, and S25DH_4_ were expected to bind a Mo-*bis*PGD, four [4Fe-4S] clusters, one [3Fe-4S] cluster, and a heme b, giving one Mo atom and 20 Fe atoms per αβγ trimer, respectively (11). The Fe content was analyzed by inductively coupled plasma atomic emission spectroscopy (ICP-AES), and the Mo content was analyzed by inductively coupled plasma mass spectroscopy (ICP-MS). The metal content of the four S25DHs varied between 0.5 and 0.8 Mo atoms and between 13.2 and 15.3 Fe atoms per αβγ trimer, which is in the range of the values determined for the wild-type enzyme (0.7 Mo atom and 16.5 Fe atoms per enzyme) (11) (Table 3).

**TABLE 3:** Kinetic parameters and metal content of heterologously produced S25DH_1_, S25DH_2_, S25DH_3_, and S25DH_4_

Enzyme and substrate	*K*_*m*_ (mM)^a,b^	*k*_*cat*_ (s^−1^)^a,b^	*k*_*cat*_/*K*_*m*_ (10^3^ M^–1^ s^−1^)^a^	Mo content^b,c^	Fe content^b,c^
S25DH_1_					
Cholest-4-en-3-one	0.39 ± 0.08	1.11 ± 0.08	2.8	0.8 ± 0.1	15.3 ± 0.5
Vitamin D_3_	0.29 ± 0.09	0.23 ± 0.01	0.79		
S25DH_2_					
Cholest-4-en-3-one	0.124 ± 0.02	0.29 ± 0.01	2.3	0.7 ± 0.1	13.5 ± 0.8
7-Dehydrocholesterol	0.123 ± 0.02	0.33 ± 0.01	2.7		
S25DH_3_					
Campest-4-en-3-one	1.84 ± 0.8	0.018 ± 0.01	0.01	0.5 ± 0.1	14.2 ± 0.5
S25DH_4_					
Cholest-4-en-3-one	0.45 ± 0.013	0.21 ± 0.02	0.46	0.7 ± 0.1	13.2 ± 0.4
Campest-4-en-3-one	0.34 ± 0.09	0.17 ± 0.02	0.50		
β-Sitost-4-en-3-one	0.12 ± 0.01	0.14 ± 0.003	1.2		

a Kinetic parameters were determined by UPLC-based enzyme assays using different concentrations of the respective substrates in the presence of 9% (wt/vol) 2-hydroxypropyl-β-cyclodextrin.

b Mean values ± standard deviations of three biological replicates are given.

c Fe content was measured by ICP-AES, and Mo content was measured by ICP-MS. The number of Mo or Fe atoms per protein is given.

### Kinetic parameters of recombinant S25DH_1_, S25DH_2_, S25DH_3_, and S25DH_4_.

The *K*_*m*_ and *k*_cat_ values of each recombinant S25DH were determined using their individual preferred substrates. Notably, the *K*_*m*_ values determined have to be regarded as apparent values, as the nearly insoluble steroid substrates were converted only in the presence of high concentrations of the solubilizing 2-hydroxypropyl-β-cyclodextrin (9%, wt/vol). The kinetic parameters are summarized in Table 3 with S25DH_1_, S25DH_2_, and S25DH_4_ showing the highest *k*_cat_/*K*_m_ values for cholest-4-en-3-one, 7-dehydrocholesterol, and β-sitost-4-en-3-one, respectively, suggesting that the respective steroids do indeed represent the preferred substrates. In the case of S25DH_3_, the low catalytic number together with the high *K*_*m*_ determined for campest-4-en-3-one indicates that the enzyme is specific for a so far unknown steroid, with campest-4-en-3-one representing the only analogue that is converted to some extent.

### Enrichment and characterization of S25DH_4_ from S. denitrificans grown with β-sitosterol.

To ascertain whether S25DH_4_ has an extended substrate spectrum toward β-sitost-4-en-3-one and campest-4-en-3-one, it was isolated from the wild-type bacteria. For this purpose, S. denitrificans was grown in a 200-liter fermenter with 3 mM β-sitosterol in a fed-batch culture, yielding 184 g cells (wet weight) (Fig. S4). Cell extracts of S. denitrificans grown with β-sitosterol converted β-sitost-4-en-3-one to the corresponding 25-OH-form at a specific activity of 0.6 nmol min^−1^ mg^−1^ (17% of the activity in recombinant T. aromatica).

10.1128/mBio.00694-18.4 FIG S4  Growth of S. denitrificans in a 200-liter fermenter with 3 mM β-sitosterol under denitrifying conditions. Download FIG S4, PDF file, 0.2 MB. Copyright © 2018 Jacoby et al. 2018 Jacoby et al. This content is distributed under the terms of the Creative Commons Attribution 4.0 International license.

**FIG 5:**
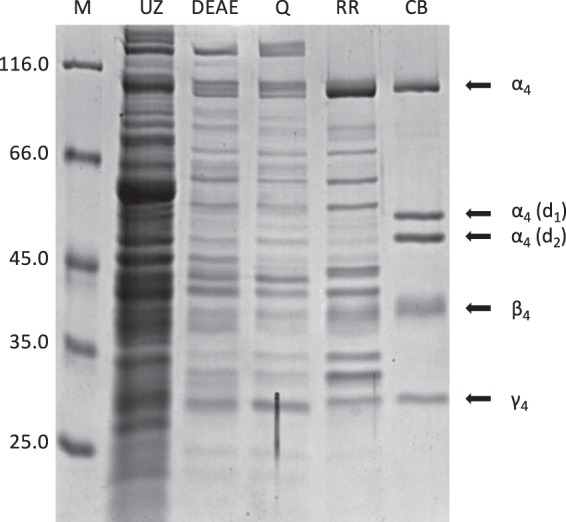
Enrichment of β-sitost-4-en-3-one converting S25DH_4_ from S. denitrificans grown with β-sitosterol and nitrate. Separation of protein fractions forming 25-OH-β-sitost-4-en-3-one by 12.5% SDS-PAGE after ultracentrifugation (16 µg of cell extract of supernatant) (UZ), chromatography on DEAE-Sepharose (24 µg protein) (DEAE), Q-Sepharose (24 µg protein) (Q), Reactive Red-agarose (20 µg) (RR), and Cibacron blue-agarose (10 µg) (CB). The arrows point to the protein bands that were identified as α_4_β_4_γ_4_ of S25DH_4_; the proteins migrating at 50 and 55 kDa were identified as degradation products of the α_4_-subunit [α_4 _(d_1_) and α_4 _(d_2_)]. Lane M contains molecular size markers (in kilodaltons).

The enrichment of β-sitost-4-en-3-one hydroxylating activity from S. denitrificans was based on the protocol described for S25DH_1_ with slight modifications (see Materials and Methods). After four chromatographic steps, five dominant protein bands were found; during SDS-PAGE, these bands eluted at 110, 55, 50, 37, and 27 kDa (Fig. 5). ESI-QTOF-MS analyses of tryptic digests identified SDENCHOL_20206 (110-, 55-, and 50-kDa bands), SDENCHOL_20205 (37-kDa band), and SDENCHOL_20204 (27-kDa band) (Table S2). This result indicates that the 50/55-kDa bands represent degradation subunits of the α_4_-subunit. Size exclusion chromatography of wild-type S25DH_4_ revealed the same values as determined for the nontagged recombinant enzyme produced in T. aromatica K172, suggesting an α_4_β_4_γ_4_ composition.

10.1128/mBio.00694-18.6 TABLE S2  Mass spectrometric analysis of enriched protein from wild-type S. denitrificans catalyzing β-sitost-4-en-3-one C-25 hydroxylation. Download TABLE S2, DOCX file, 0.01 MB. Copyright © 2018 Jacoby et al. 2018 Jacoby et al. This content is distributed under the terms of the Creative Commons Attribution 4.0 International license.

S25DH_4_ was 55-fold enriched from S. denitrificans with a maximal specific activity for β-sitost-4-en-3-one conversion of 33 nmol min^−1^ mg^−1^. Both wild-type and recombinant S25DH_4_ exhibited the identical substrate preference and converted β-sitost-4-en-3-one, cholest-4-en-3-one, and campest-4-en-3-one (Table 3). In summary, the S25DH_4_ composed of the α_4_β_4_γ_4_ subunits has an extended substrate specificity for β-sitost-4-en-3-one (and campest-4-en-3-one); as shown for S25DH_1_, the specific activity of S25DH_4_ in cell extracts was around sixfold higher when heterologously produced in T. aromatica compared to the wild-type S25DH_4_.

## DISCUSSION

So far, a number of DMSOR family members have been produced by homologously or heterologously expressing their corresponding genes (27–34). In contrast, attempts to produce the phylogenetically related, complex heterotrimeric molybdenum-iron-sulfur/heme b-containing alkyl chain hydroxylases of the class II DMSOR family in an active form have failed so far. In this work we now provide a tool for their recombinant production in highly active forms as demonstrated for the example of four S25DH isoenzymes with differing substrate specificities. The expression platform opens the door for studying other alkyl chain-hydroxylating members of the DMSOR family comprising enzymes specifically forming tertiary (S25DHs), secondary (ethylbenzene DH) (17, 18), and primary (*p*-cymene DH) (19) alcohols, and many others of unknown function. Notably, production of highly active enzymes in the presence of SdhD required the expression of all three functional genes.

Production in *Thauera *aromatica yielded crude extract activities that in the case of S25DH_1_ and S25DH_4_ were sixfold higher than in cell extracts from the parental Sterolibacterium denitrificans after growth with the preferred substrates cholesterol (S25DH_1_) and β-sitosterol (S25DH_4_). The apparent overexpression largely facilitated and shortened the enrichment procedure by using only two chromatographic steps, which in turn greatly reduced to a minimum the generally observed degradation of the active site α-subunit. As a further advantage, the specific activities of the isolated enzymes of S25DH_1_ and S25DH_4_ were twofold higher than after purification from the wild type. Finally, while purification from the wild-type bacteria always yielded mixtures of different isoenzymes, the T. aromatica expression platform produced a preparation of a single S25DH. In summary, production in T. aromatica largely enhanced yield, specific activity, and specificity.

The results obtained now allow for the unambiguous assignment of substrate specificities to individual S25DH isoenzymes. The prototypical S25DH_1_, originally isolated from S. denitrificans cells grown with cholesterol (11), used cholest-4-en-3-one as the preferred substrate. The S25DH_2_ has an extended substrate spectrum toward 7-dehydrocholesterol and S25DH_4_ for the phytosterol-derived β-sitost-4-en-3-one and campest-4-en-3-one with (*R*)-configured ethyl and methyl branches at C-24, respectively. The function of S25DH_3_ appears to be less clear, as it showed only minor activity with campest-4-en-3-one indicating that the natural substrate is still at issue. None of the four S25DHs showed significant activity with sterols containing a Δ22,23 double bond in the side chain such as ergosterol, brassica-4-en-3-one, or stigmast-4-en-3-one, although ergosterol and stigmasterol are growth substrates. This finding suggests that either one of the four remaining S25DHs is involved in their conversion, or a more likely alternative is that the double bond needs to be reduced or otherwise converted prior to C-25 hydroxylation. As the abundance of S25DH_3_ in cells grown with ergosterol was clearly increased (8), it is likely that ergosterol is first converted to a so far unknown intermediate that then serves as the substrate for S25DH_3_. However, reduction of the nonactivated Δ22,23 double bond can hardly be achieved in the absence of oxygen with physiological electron donors. The function of S25DH_5_, S25DH_6_, S25DH_7_, and S25DH_8_ needs to be elucidated in the future using the system established in this work. S25DH_7_ has recently been proposed to be involved in the conversion of 25- to 26-OH-cholest-4-en-3-one. It is striking that the α-subunit of putative S25DH_8_ is more closely related to that of *p*-cymene dehydrogenase and to an S25DH-like enzyme from Thauera terpenica (see Fig. S1 in the supplemental material), suggesting that it may play a role in hydroxylating a nonsteroidal isoprenoid compound.

This work demonstrated that of the four S25DH isoenzymes investigated in this work, S25DH_1_ is the optimal catalyst for VD_3_ hydroxylation to the clinically relevant 25-OH-VD_3_ (22). This reaction is of considerable biotechnological potential, as it has several advantages in comparison to multistep chemical (35) or oxygenase- and electron donor-dependent 25-OH-VD_3_ synthesis procedures (36–40). The established expression platform overcomes previously identified limitations of S25DH-catalyzed 25-OH-VD_3_ synthesis, which have largely prevented biotechnological application so far. First, the easy and rapid enrichment procedure minimizes degradation of the α-subunit as always observed during purification from the wild-type strain. As a result, the specific activities of the enriched S25DHs are around twofold higher in the recombinant than in the wild-type strain. Second, S25DH_1_ was produced to a 6.4-fold-higher extent in *T.* aromatica than in the wild-type bacteria, resulting in the corresponding increase of the VD_3_ conversion rate in crude extracts. As a consequence, enzymatic 25-OH-VD_3_ synthesis by crude extracts is drastically shortened and can now be accomplished even under aerobic conditions without a significant loss of activity. Third, due to the lack of enzymes catalyzing downstream reactions of anaerobic steroid degradation in T. aromatica, the addition of AgNO_3_, the established inhibitor of these reactions, is now dispensable when using crude extracts for VD_3_ conversion.

In summary, the expression platform established in this work provides not only easy access to previously nonstudied members of the alkyl chain-hydroxylating DMSOR family members, it also allows for previously hardly achievable mechanistic studies (e.g., site-directed mutagenesis) and applied studies (25-OH-VitD_3_ synthesis) of a catalytically versatile class of molybdenum enzymes.

## MATERIALS AND METHODS

### Chemicals and bacterial strains.

The chemicals used were of analytic grade. Sterolibacterium denitrificans Chol-1S^T^ (DSMZ 13999) and Thauera aromatica K172 (DSMZ 6984) were obtained from the Deutsche Sammlung für Mikroorganismen und Zellkulturen (DSMZ, Braunschweig, Germany). *Azoarcus* sp. strain CIB (CECT 5669) was obtained from the Spanish Type Culture Collection (Valencia, Spain), E. coli BL21(DE3) was from New England Biolabs (Frankfurt, Germany) and E. coli Top 10 was from Thermo Fisher Scientific (Carlsbad, CA).

### Synthesis of steroidal substrates.

Substrates for S25DHs were produced from commercially available steroid precursors (β-sitosterol, campesterol, brassicasterol, and stigmasterol) using cholesterol oxidase from *Streptomyces* sp. according to the manufacturer’s protocol (Sisco Research Laboratories Pvt. Ltd.). The enzyme assays contained 50 mM Tris/PO_4_ buffer (pH 7.5), 9% 2-hydroxypropyl-β-cyclodextrin (wt/vol), 2.5 mM NAD^+^, 1 mM substrate, 2 mM MgCl_2_, cholesterol oxidase, and catalase. Products were extracted and purified by high-performance liquid chromatography (HPLC) as described elsewhere (11). Samples were lyophilized and diluted in 1,4-dioxane.

### Culture conditions and preparation of cell extracts.

S. denitrificans was cultivated under denitrifying conditions in mineral medium with steroid substrates as described previously (7). Cells were harvested in the late exponential growth phase by centrifugation (8,000 × *g*, 20 min, 4°C). During large-scale cultivation with β-sitosterol (3 mM) in a 200-liter fermenter, nitrate was discontinuously added in 10 mM steps. T. aromatica K172 and *Azoarcus* sp. CIB expressing genes encoding S25DHs were cultivated under denitrifying conditions using a phosphate-buffered medium with benzoate as the carbon source at 30°C as described previously (41). Cells were harvested anaerobically by centrifugation (8,000 × *g*, 20 min, 4°C) in the late exponential phase. Frozen cells were suspended in 2 volumes of lysis buffer (wt/vol) containing 20 mM Tris/PO_4_ (pH 7.0), 0.1 mg DNase I, 1 mM dithiothreitol (DTT), and 0.02% (wt/vol) Tween 20. Cells were lysed by passage through a French pressure cell at 137 MPa, solubilized for 3 h, and centrifuged at 150,000 × *g* for 1.5 h at 4°C. The anaerobically prepared supernatant was used for enzyme purification.

### Enzyme assays.

Enzyme assays were anaerobically carried out at 30°C in 100 mM Tris/PO_4_ buffer (pH 7.0) with 0.5 mM steroid substrates (25 mM concentrated in 1,4-dioxane), 10 mM K_3_[Fe(CN)_6_], and 9% 2-hydroxypropyl-β-cyclodextrin (wt/vol) following substrate consumption and product formation by ultrahigh-performance liquid chromatography (UPLC) as described previously (11, 22). For the conversion of cholest-4-en-3-one with cell extracts of S. denitrificans, 0.5 mM AgNO_3_ were added to prevent further oxidation of the substrate.

### Heterologous gene expression and S25DH production.

The genes encoding the αβγ-subunits together with the chaperone (SdhD) were amplified by PCR from S. denitrificans genomic DNA as the template, using primers suitable for T4 ligation (see Table S3 in the supplemental material). Genes encoding γ_3_β_3_-subunits were amplified together by using S25DHγ_3_β_3__for (for stands for forward) and S25DHγ_3_β_3__rev (rev stands for reverse) primers, and the resulting 1.8-kb DNA fragment was ClaI/HindIII doubly digested; the genes encoding α_1_ and α_3_ subunits were amplified by using S25DHα_1-3__for and S25DHα_1-3__rev primers, generating a 2.9-kb DNA fragment that was HindIII/SpeI doubly digested. The gene encoding SdhD was amplified using SdhD_for and SdhD_rev primers, generating a 0.9-kb DNA fragment that was SpeI/XbaI doubly digested. All fragments were sequentially cloned into the broad-host-range vector pIZ1016 (Gm^r^, *ori*pBBR1, Mob^+^, *lacZ*α, *Ptac*/*lacI*^q^), a derivative of pBBR1MCS-5, bearing the *tac* promoter and *lacI*^q^ regulatory gene from pMM40 (42), and transformed in E. coli NEB5α (New England Biolabs GmbH) according to the manufacturer’s protocol. Genes encoding the α_4_β_4_γ_4_ SdhD components of S25DH_4_ were amplified with specific primers suitable for Gibson assembly (Table S3) using the Gibson Assembly master mix (New England Biolabs GmbH) at 50°C for 1 h. The following steps are described for T. aromatica but were also similarly applied for *Azoarcus* sp. CIB. The genes were transformed into T. aromatica cells by electroporation as follows: T. aromatica was cultivated on phosphate-buffered medium (25 mM acetate) at 30°C until it reached an optical density at 578 nm (OD_578_) of 0.4 to 0.6, centrifuged (4,500 × *g*, 15 min, 4°C), and washed twice in 20% volume ice-cold 1 mM morpholinepropanesulfonic acid (MOPS) buffer. Centrifuged cells (4,500 × *g*, 15 min, 4°C) were suspended in 0.5% volume ice-cold 1 mM MOPS–15% glycerol (wt/vol). Cells were immediately used for transformation at 1.7 kV (Eppendorf Eporator) and incubated in medium at 30°C for 4 h before plated on selection medium (0.8% Gelrite, 0.025% MgSO_4 _⋅_ _7H_2_O, 25 mM acetate, and 20 µg ml^−1^ gentamicin). Cultivation was carried out aerobically at 30°C until colonies were formed. For gene expression, cells were grown under denitrifying conditions in phosphate-buffered medium with benzoate as the carbon source (see above) supplemented with 20 µg ml^−1^ gentamicin. Expression of S25DH genes was fostered by adding 1 mM IPTG at an optical density of 0.7. After subsequent growth for 48 h, cells were harvested anaerobically in the late exponential phase. For gene expression of S25DH_1_ in E. coli BL21 and E. coli Top 10, cells were cultivated anaerobically (25 mM nitrate) at 30°C in M4 minimal medium and aerobically in 2× YT medium at 20°C complemented with 20 µg ml^−1^ gentamicin.

10.1128/mBio.00694-18.7 TABLE S3  Oligonucleotide primers used for heterologous production of steroid C-25 dehydrogenases. Download TABLE S3, DOCX file, 0.01 MB. Copyright © 2018 Jacoby et al. 2018 Jacoby et al. This content is distributed under the terms of the Creative Commons Attribution 4.0 International license.

### Enrichment of recombinant proteins from T. aromatica.

After ultracentrifugation (150,000 × *g*, 1.5 h, 4°C), the soluble protein fraction was applied to a DEAE-Sepharose column under anaerobic conditions (50 ml; GE Healthcare) at 5 ml min^−1^ and washed with buffer A (20 mM Tris/PO_4_ [pH 7.0], 1 mM DTT, and 0.02% [wt/vol] Tween 20). The soluble protein fraction was eluted by increasing the amount of buffer B (20 mM Tris/PO_4_ [pH 7.0], 1 mM DTT, 0.02% [wt/vol] Tween 20, and 500 mM KCl) from 12% to 16%. Active fractions were concentrated (30-kDa cutoff membrane), diluted in 10 volumes of buffer C (20 mM Tris/morpholineethanesulfonic acid [MES] [pH 6.0], 1 mM DTT), and applied to a Reactive Red 120 column (50 ml; GE Healthcare) at 5 ml min^−1^. The active fractions were elute with an increasing gradient of buffer D from 0 to 100% (Tris/PO_4_ [pH 8.0], 1 mM DTT) in 20% steps. S25DH_1_, S25DH_2_, S25DH_3_, and S25DH_4_ eluted at pH values of approximately 6.5, 6.7, 7.5, and 7.8, respectively.

### Purification of S25DH_4_ from S. denitrificans.

Soluble proteins were applied to a DEAE Sepharose column at 1.5 ml min^−1^ and washed with buffer A. Column-bound proteins were enriched using a stepwise gradient (10%) of buffer B from 10% to 20%. The active fraction was diluted 1:3 with buffer A and applied to a Q-Sepharose column (20 ml; GE Healthcare) at 0.5 ml min^−1^. The active fraction was eluted by a gradient of buffer C_2_ (20 mM MES/Tris [pH 6.0], 1 mM DTT, 0.02% Tween 20, and 500 mM KCl) from 10% to 20%. The active fractions were diluted in 1:10 in buffer C and applied to a Reactive Red 120 column at 0.5 ml min^−1^. The active fractions were eluted by increasing the concentration of buffer C_2_ from 80% to 100%. Activity-containing fractions were concentrated, desalted, and screened for activity. Active fractions were applied to a Cibacron blue-Sepharose column (1 ml; GE Healthcare) at 0.5 ml min^−1^ using buffer C. The active fractions were eluted by increasing the concentration of buffer D in 20% steps from 80% to 100%. The active fractions were pooled and concentrated (30-kDa cutoff membrane).

### Determination of Mo and Fe content by ICP-MS/ICP-AES.

Inductively coupled plasma mass spectroscopy (ICP-MS)/inductively coupled plasma atomic emission spectroscopy (ICP-AES) analyses were conducted to determine the Mo and Fe content of heterologously produced S25DHs as described previously (43).

### Gel filtration.

The native molecular weight of steroid C-25 dehydrogenases was analyzed by size exclusion chromatography on a Superdex 200 column (GE Healthcare) at 0.5 ml min^−1^ in 50 mM Tris-HCl (pH 7.0)–150 mM NaCl. Proteins used for calibration were thyroglobulin (669 kDa), apoferritin (443 kDa), alcohol dehydrogenase (150 kDa), carbonic anhydrase (29 kDa), and cytochrome *c* (12.4 kDa).

### Protein identification by mass spectrometry.

Proteins were identified by excising the bands of interest from an SDS-PAGE. After in-gel digestion with trypsin (Sigma-Aldrich), the resulting peptides were separated by UPLC and identified by using a Synapt G2-Si high-definition mass spectrometry (HDMS) electrospray ionization quadrupole time of flight (ESI-QTOF) system (Waters) as described previously (44).

## References

[1] Dufourc EJ 2008 Sterols and membrane dynamics. J Chem Biol 1:63–77. doi:10.1007/s12154-008-0010-6. 19568799 PMC2698314

[2] Nes WD 2011 Biosynthesis of cholesterol and other sterols. Chem Rev 111:6423–6451. doi:10.1021/cr200021m. 21902244 PMC3191736

[3] Barbosa MO, Moreira NFF, Ribeiro AR, Pereira MFR, Silva AMT 2016 Occurrence and removal of organic micropollutants: an overview of the watch list of EU Decision 2015/495. Water Res 94:257–279. doi:10.1016/j.watres.2016.02.047. 26967909

[4] Ting YF, Praveena SM 2017 Sources, mechanisms, and fate of steroid estrogens in wastewater treatment plants: a mini review. Environ Monit Assess 189:178. doi:10.1007/s10661-017-5890-x. 28342046

[5] Bergstrand LH, Cardenas E, Holert J, Van Hamme JD, Mohn WW 2016 Delineation of steroid-degrading microorganisms through comparative genomic analysis. mBio 7:e00166. doi:10.1128/mBio.00166-16. 26956583 PMC4810484

[6] Kieslich K 1985 Microbial side chain degradation of sterols. J Basic Microbiol 25:461–474. doi:10.1002/jobm.3620250713. 3903107

[7] Tarlera S, Denner EB 2003 *Sterolibacterium denitrificans* gen. nov., sp. nov., a novel cholesterol-oxidizing, denitrifying member of the beta-Proteobacteria. Int J Syst Evol Microbiol 53:1085–1091. doi:10.1099/ijs.0.02039-0. 12892131

[8] Warnke M, Jacoby C, Jung T, Agne M, Mergelsberg M, Starke R, Jehmlich N, von Bergen M, Richnow HH, Brüls T, Boll M 2017 A patchwork pathway for oxygenase-independent degradation of side chain containing steroids. Environ Microbiol 19:4684–4699. doi:10.1111/1462-2920.13933. 28940833

[9] Yang FC, Chen YL, Tang SL, Yu CP, Wang PH, Ismail W, Wang CH, Ding JY, Yang CY, Yang CY, Chiang YR 2016 Integrated multi-omics analyses reveal the biochemical mechanisms and phylogenetic relevance of anaerobic androgen biodegradation in the environment. ISME J 10:1967–1983. doi:10.1038/ismej.2015.255. 26872041 PMC5029156

[10] Chiang YR, Ismail W, Müller M, Fuchs G 2007 Initial steps in the anoxic metabolism of cholesterol by the denitrifying *Sterolibacterium denitrificans*. J Biol Chem 282:13240–13249. doi:10.1074/jbc.M610963200. 17307741

[11] Dermer J, Fuchs G 2012 Molybdoenzyme that catalyzes the anaerobic hydroxylation of a tertiary carbon atom in the side chain of cholesterol. J Biol Chem 287:36905–36916. doi:10.1074/jbc.M112.407304. 22942275 PMC3481293

[12] Wang PH, Lee TH, Ismail W, Tsai CY, Lin CW, Tsai YW, Chiang YR 2013 An oxygenase-independent cholesterol catabolic pathway operates under oxic conditions. PLoS One 8:e66675. doi:10.1371/journal.pone.0066675. 23826110 PMC3691188

[13] Warnke M, Jung T, Jacoby C, Agne M, Feller FM, Philipp B, Seiche W, Breit B, Boll M 2018 Functional characterization of three specific acyl-coenzyme A synthetases involved in anaerobic cholesterol degradation in *Sterolibacterium denitrificans* Chol1S. Appl Environ Microbiol 84:e02721-17. doi:10.1128/AEM.02721-17. 29374035 PMC5861837

[14] Wang PH, Leu YL, Ismail W, Tang SL, Tsai CY, Chen HJ, Kao AT, Chiang YR 2013 Anaerobic and aerobic cleavage of the steroid core ring structure by *Steroidobacter denitrificans*. J Lipid Res 54:1493–1504. doi:10.1194/jlr.M034223. 23458847 PMC3622341

[15] Wang PH, Yu CP, Lee TH, Lin CW, Ismail W, Wey SP, Kuo AT, Chiang YR 2014 Anoxic androgen degradation by the denitrifying bacterium *Sterolibacterium denitrificans* via the 2,3-seco pathway. Appl Environ Microbiol 80:3442–3452. doi:10.1128/AEM.03880-13. 24657867 PMC4018845

[16] Crowe AM, Casabon I, Brown KL, Liu J, Lian J, Rogalski JC, Hurst TE, Snieckus V, Foster LJ, Eltis LD 2017 Catabolism of the last two steroid rings in *Mycobacterium tuberculosis* and other bacteria. mBio 8:e00321-17. doi:10.1128/mBio.00321-17. 28377529 PMC5380842

[17] Kniemeyer O, Heider J 2001 Ethylbenzene dehydrogenase, a novel hydrocarbon-oxidizing molybdenum/iron-sulfur/heme enzyme. J Biol Chem 276:21381–21386. doi:10.1074/jbc.M101679200. 11294876

[18] Johnson HA, Pelletier DA, Spormann AM 2001 Isolation and characterization of anaerobic ethylbenzene dehydrogenase, a novel Mo-Fe-S enzyme. J Bacteriol 183:4536–4542. doi:10.1128/JB.183.15.4536-4542.2001. 11443088 PMC95348

[19] Strijkstra A, Trautwein K, Jarling R, Wöhlbrand L, Dörries M, Reinhardt R, Drozdowska M, Golding BT, Wilkes H, Rabus R 2014 Anaerobic activation of p-cymene in denitrifying betaproteobacteria: methyl group hydroxylation versus addition to fumarate. Appl Environ Microbiol 80:7592–7603. doi:10.1128/AEM.02385-14. 25261521 PMC4249252

[20] Grimaldi S, Schoepp-Cothenet B, Ceccaldi P, Guigliarelli B, Magalon A 2013 The prokaryotic Mo/W-bisPGD enzymes family: a catalytic workhorse in bioenergetic. Biochim Biophys Acta 1827:1048–1085. doi:10.1016/j.bbabio.2013.01.011. 23376630

[21] Rugor A, Wójcik-Augustyn A, Niedzialkowska E, Mordalski S, Staroń J, Bojarski A, Szaleniec M 2017 Reaction mechanism of sterol hydroxylation by steroid C25 dehydrogenase—homology model, reactivity and isoenzymatic diversity. J Inorg Biochem 173:28–43. doi:10.1016/j.jinorgbio.2017.04.027. 28482186

[22] Warnke M, Jung T, Dermer J, Hipp K, Jehmlich N, von Bergen M, Ferlaino S, Fries A, Müller M, Boll M 2016 25-Hydroxyvitamin D3 synthesis by enzymatic steroid side-chain hydroxylation with water. Angew Chem Int Ed Engl 55:1881–1884. doi:10.1002/anie.201510331. 26695374

[23] Tian XQ, Holick MF 1995 Catalyzed thermal isomerization between previtamin D_3_ and vitamin D3 via beta-cyclodextrin complexation. J Biol Chem 270:8706–8711. doi:10.1074/jbc.270.15.8706. 7721775

[24] Rugor A, Tataruch M, Staroń J, Dudzik A, Niedzialkowska E, Nowak P, Hogendorf A, Michalik-Zym A, Napruszewska DB, Jarzębski A, Szymańska K, Białas W, Szaleniec M 2017 Regioselective hydroxylation of cholecalciferol, cholesterol and other sterol derivatives by steroid C25 dehydrogenase. Appl Microbiol Biotechnol 101:1163–1174. doi:10.1007/s00253-016-7880-2. 27726023

[25] Martín-Moldes Z, Zamarro MT, Del Cerro C, Valencia A, Gómez MJ, Arcas A, Udaondo Z, García JL, Nogales J, Carmona M, Díaz E 2015 Whole-genome analysis of *Azoarcus* sp. strain CIB provides genetic insights to its different lifestyles and predicts novel metabolic features. Syst Appl Microbiol 38:462–471. doi:10.1016/j.syapm.2015.07.002. 26259823

[26] Anders HJ, Kaetzke A, Kampfer P, Ludwig W, Fuchs G 1995 Taxonomic position of aromatic-degrading denitrifying pseudomonad strains K 172 and KB 740 and their description as new members of the genera *Thauera*, as *Thauera aromatica* sp. nov., and *Azoarcus*, as *Azoarcus evansii* sp. nov., respectively, members of the beta subclass of the *Proteobacteria*. Int J Syst Bacteriol 45:327–333. doi:10.1099/00207713-45-2-327. 7537067

[27] Chu S, Zhang D, Wang D, Zhi Y, Zhou P 2017 Heterologous expression and biochemical characterization of assimilatory nitrate and nitrite reductase reveals adaption and potential of *Bacillus megaterium* NCT-2 in secondary salinization soil. Int J Biol Macromol 101:1019–1028. doi:10.1016/j.ijbiomac.2017.04.009. 28389402

[28] Hartmann T, Leimkühler S 2013 The oxygen-tolerant and NAD+-dependent formate dehydrogenase from *Rhodobacter capsulatus* is able to catalyze the reduction of CO2 to formate. FEBS J 280:6083–6096. doi:10.1111/febs.12528. 24034888

[29] Hilton JC, Temple CA, Rajagopalan KV 1999 Re-design of *Rhodobacter sphaeroides* dimethyl sulfoxide reductase. Enhancement of adenosine N1-oxide reductase activity. J Biol Chem 274:8428–8436. doi:10.1074/jbc.274.13.8428. 10085074

[30] Kappler U, McEwan AG 2002 A system for the heterologous expression of complex redox proteins in *Rhodobacter capsulatus*: characterisation of recombinant sulphite:cytochrome c oxidoreductase from *Starkeya novella*. FEBS Lett 529:208–214. doi:10.1016/S0014-5793(02)03344-6. 12372602

[31] Malasarn D, Keeffe JR, Newman DK 2008 Characterization of the arsenate respiratory reductase from *Shewanella* sp. strain ANA-3. J Bacteriol 190:135–142. doi:10.1128/JB.01110-07. 17951391 PMC2223751

[32] Pollock VV, Conover RC, Johnson MK, Barber MJ 2002 Bacterial expression of the molybdenum domain of assimilatory nitrate reductase: production of both the functional molybdenum-containing domain and the nonfunctional tungsten analog. Arch Biochem Biophys 403:237–248. doi:10.1016/S0003-9861(02)00215-1. 12139973

[33] Tenbrink F, Schink B, Kroneck PM 2011 Exploring the active site of the tungsten, iron-sulfur enzyme acetylene hydratase. J Bacteriol 193:1229–1236. doi:10.1128/JB.01057-10. 21193613 PMC3067604

[34] Wu SY, Rothery RA, Weiner JH 2015 Pyranopterin coordination controls molybdenum electrochemistry in *Escherichia coli* nitrate reductase. J Biol Chem 290:25164–25173. doi:10.1074/jbc.M115.665422. 26297003 PMC4599019

[35] Zhu GD, Okamura WH 1995 Synthesis of vitamin D (calciferol). Chem Rev 95:1877–1952. doi:10.1021/cr00038a007.

[36] Hayashi K, Yasuda K, Sugimoto H, Ikushiro S, Kamakura M, Kittaka A, Horst RL, Chen TC, Ohta M, Shiro Y, Sakaki T 2010 Three-step hydroxylation of vitamin D3 by a genetically engineered CYP105A1: enzymes and catalysis. FEBS J 277:3999–4009. doi:10.1111/j.1742-4658.2010.07791.x 20731719

[37] Kang DJ, Im JH, Kang JH, Kim KH 2015 Bioconversion of vitamin D3 to calcifediol by using resting cells of *Pseudonocardia* sp. Biotechnol Lett 37:1895–1904. doi:10.1007/s10529-015-1862-9. 25994584

[38] Sakaki T, Sugimoto H, Hayashi K, Yasuda K, Munetsuna E, Kamakura M, Ikushiro S, Shiro Y 2011 Bioconversion of vitamin D to its active form by bacterial or mammalian cytochrome P450. Biochim Biophys Acta 1814:249–256. doi:10.1016/j.bbapap.2010.07.014. 20654743

[39] Yasuda K, Endo M, Ikushiro S, Kamakura M, Ohta M, Sakaki T 2013 UV-dependent production of 25-hydroxyvitamin D2 in the recombinant yeast cells expressing human CYP2R1. Biochem Biophys Res Commun 434:311–315. doi:10.1016/j.bbrc.2013.02.124. 23548573

[40] Yasutake Y, Nishioka T, Imoto N, Tamura T 2013 A single mutation at the ferredoxin binding site of P450 Vdh enables efficient biocatalytic production of 25-hydroxyvitamin D3. Chembiochem 14:2284–2291. doi:10.1002/cbic.201300386. 24115473

[41] Tschech A, Fuchs G 1987 Anaerobic degradation of phenol by pure cultures of newly isolated denitrifying pseudomonads. Arch Microbiol 148:213–217. doi:10.1007/BF00414814. 3675113

[42] Kovach ME, Elzer PH, Hill DS, Robertson GT, Farris MA, Roop RM, Peterson KM 1995 Four new derivatives of the broad-host-range cloning vector pBBR1MCS, carrying different antibiotic-resistance cassettes. Gene 166:175–176. doi:10.1016/0378-1119(95)00584-1. 8529885

[43] Weinert T, Huwiler SG, Kung JW, Weidenweber S, Hellwig P, Stärk HJ, Biskup T, Weber S, Cotelesage JJ, George GN, Ermler U, Boll M 2015 Structural basis of enzymatic benzene ring reduction. Nat Chem Biol 11:586–591. doi:10.1038/nchembio.1849. 26120796

[44] Mergelsberg M, Willistein M, Meyer H, Stärk HJ, Bechtel DF, Pierik AJ, Boll M 2017 Phthaloyl-CoA decarboxylase from *Thauera chlorobenzoica*: the prenylated flavin, K^+^, and Fe^2+^-dependent key enzyme of anaerobic phthalate degradation. Environ Microbiol 19:3734–3744. doi:10.1111/1462-2920.13875. 28752942

