# Supplementary material for: Four Molybdenum-Dependent Steroid C-25 Hydroxylases: Heterologous Overproduction, Role in Steroid Degradation, and Application for 25-Hydroxyvitamin D3 Synthesis
Source: mBio. 2018 Jun 19;9(3):e00694-18. doi: 10.1128/mBio.00694-18 (PMC6016249; doi:10.1128/mBio.00694-18)
Supplement: FIG S1 [file mbo003183935sf1.pdf]

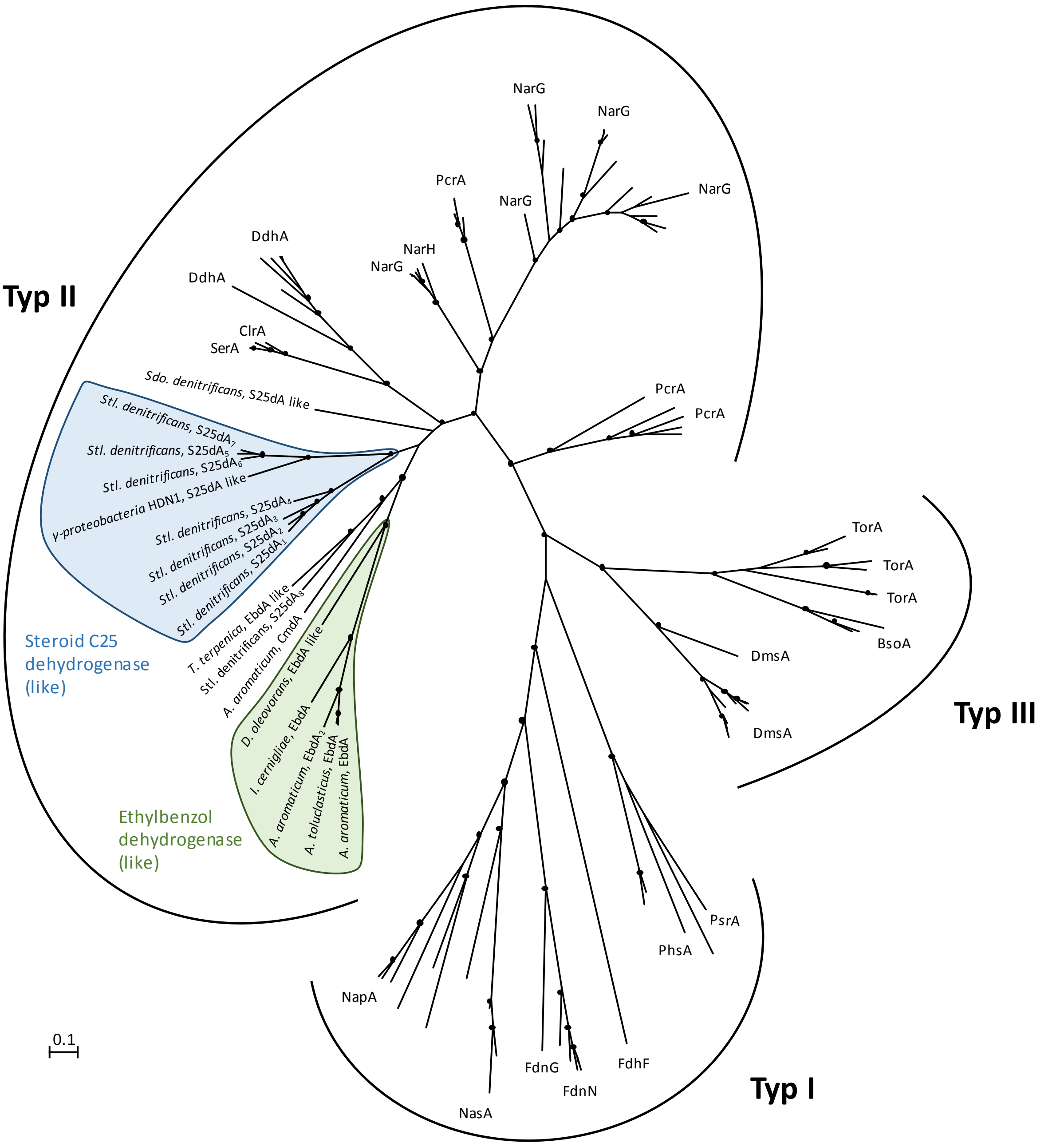

Fig S1 Phylogenetic tree of active site α-subunits from S25DH-like enzymes inside the DMSOR family of MoCo-containing enzymes. The affiliation of the enzymes to the type I – III molybdenum enzymes is marked. The catalytic subunits of Steroid C25 like dehydrogenases (S25dA like) and ethylbenzol like dehydrogenases (ebdA like) are highlighted. The phylogenetic tree was created using the Maximum Likelihood method based on the Poisson correction model. Bootstrap values (1000) higher than 75% are marked by dots. Abbreviations: (S25dA) – Steroid C25 dehydrogenase α subunit; (EbdA) - ethylbenzene dehydrogenase α subunit; (CmdA) – p-cymene dehydrogenase α subunit; (SerA) – Selenate reductase α subunit; (ClrA) – chlorate reductase α subunit; (DdhA) – Dimethyl sulfide dehydrogenase α subunit; (NarG/H) – respiratory nitrate reductase α subunit; (PcrA) – perchlorate reductase α subunit; (TorA) – Trimethylamin-N-oxide reductase α subunit; (BsoA) – biotin sulfoxide reductase α subunit; (DmsA) – Dimethyl sulfoxide reductase α subunit; (PsrA) – polysulfide reductase α subunit; (PhsA) – thiosulfate reductase α subunit; (FdnN/G) – formate dehydrogenase α subunit; (NasA) – assimilatoric nitrate reductase α subunit; (NapA) – periplasmatic nitrate reductase α subunit. (*Stl. denitrificans*) – *Sterolibacterium denitrificans*; (*Sdo. denitrificans*) – *Steroidobacter denitrificans*; (*A. aromaticum*) – *Aromatoleum aromaticum*; (*A. toluclasticus*) – *Aromatoleum toluclasticus*; (*D. oleovorans*) – *Desulfococcusoleovorans*; (*T. terpenica*) – *Thauera terpenica*; (*I. cernigliae*) – *Immundisolibacter cernigliae*.
