# Supplementary material for: Four Molybdenum-Dependent Steroid C-25 Hydroxylases: Heterologous Overproduction, Role in Steroid Degradation, and Application for 25-Hydroxyvitamin D3 Synthesis
Source: mBio. 2018 Jun 19;9(3):e00694-18. doi: 10.1128/mBio.00694-18 (PMC6016249; doi:10.1128/mBio.00694-18)
Supplement: FIG S2 [file mbo003183935sf2.pdf]

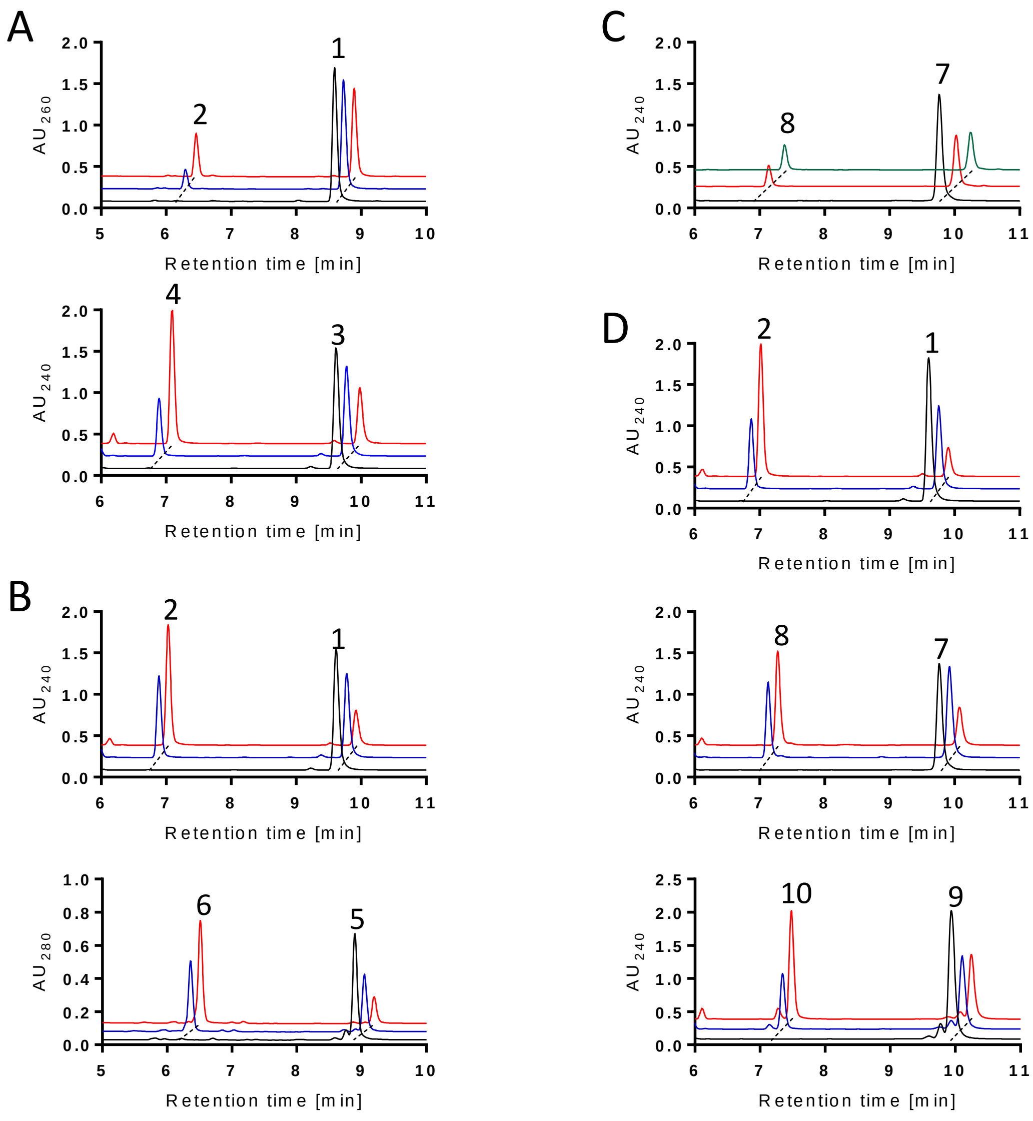

Fig S2 UPLC chromatograms showing the conversion of various steroid substrates by different recombinant S25DHs from *Stl. denitrificans* heterologously produced in *T. aromatica*. **A**, S25DH1 (0.5 mg ml^{-1}); **B**, S25DH2 (0.5 mg ml^{-1}); **C**, S25DH3 (2 mg ml^{-1}), and **D**, S25DH4 (1 mg mL^{-1}). Black lines: 0 min; blue lines: 30 min; red lines: 120 min; green lines: 360 min of incubation. The following substrates were added at 0.5 mM: cholest-4-en-3-one (1), vitamin D3 (VD3) (3), 7-dehydrocholesterol (5), campest-4-en-3-one (7) and sitost-4-en-3-one (9). They were converted to 25-OH-cholest-4-en-3-one (2), 25-OH-VD3 (4), 25-OH-7-dehydrocholesterol (6), 25-OH-campest-4-en-3-one (8) and 25-OH-sitost-4-en-3-one (10).
