# Supplementary material for: Four Molybdenum-Dependent Steroid C-25 Hydroxylases: Heterologous Overproduction, Role in Steroid Degradation, and Application for 25-Hydroxyvitamin D3 Synthesis
Source: mBio. 2018 Jun 19;9(3):e00694-18. doi: 10.1128/mBio.00694-18 (PMC6016249; doi:10.1128/mBio.00694-18)
Supplement: FIG S3 [file mbo003183935sf3.pdf]

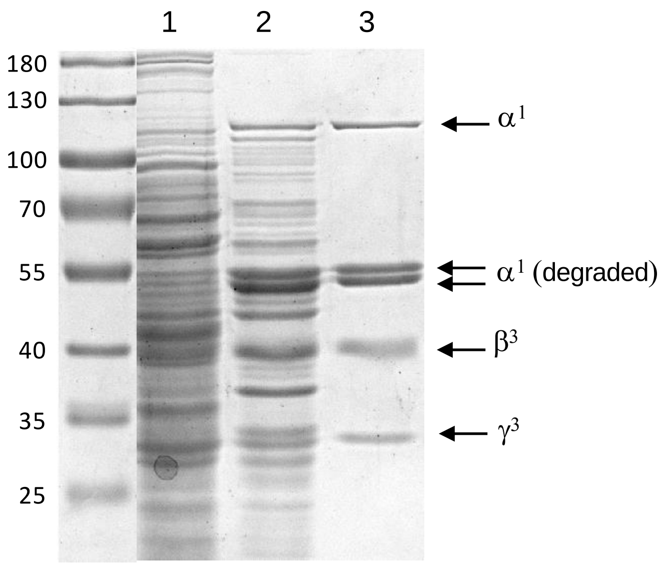

Fig S3. SDS-Page (12.5%) of the active fractions during enrichment of S25DH1 ($\alpha_1\beta_3\gamma_3$) in *Azoarcus sp.* CIB. Lane 1, 150.000 *g* supernatant (20 µg protein); Lane 2, DEAE-Sepharose (10 µg); Lane 3, Reactive Red affinity chromatography fraction (5 µg). The arrows point to the respective three subunits.
