# Supplementary material for: Four Molybdenum-Dependent Steroid C-25 Hydroxylases: Heterologous Overproduction, Role in Steroid Degradation, and Application for 25-Hydroxyvitamin D3 Synthesis
Source: mBio. 2018 Jun 19;9(3):e00694-18. doi: 10.1128/mBio.00694-18 (PMC6016249; doi:10.1128/mBio.00694-18)
Supplement: TABLE S1 [file mbo003183935st1.docx]

**Table S1** Mass spectrometric analysis of S25DH_1_ from *Stl. denitrificans* heterologously produced in *T. aromatica* K172. The excised bands correspond to those in Fig. 3, left panel.

| **Mass of excised SDS protein band** | **Gene product identified** | **Matched peptides** | **Sequence coverage** |
| --- | --- | --- | --- |
| 110 | SDENCHOL_20804 (α_1_) | 55 | 63% |
| 40 | SDENCHOL_20461 (β_3_) | 18 | 53% |
| 25 | SDENCHOL_20462 (γ_3_) | 6 | 33% |
