# Supplementary material for: Four Molybdenum-Dependent Steroid C-25 Hydroxylases: Heterologous Overproduction, Role in Steroid Degradation, and Application for 25-Hydroxyvitamin D3 Synthesis
Source: mBio. 2018 Jun 19;9(3):e00694-18. doi: 10.1128/mBio.00694-18 (PMC6016249; doi:10.1128/mBio.00694-18)
Supplement: FIG S4 [file mbo003183935sf4.pdf]

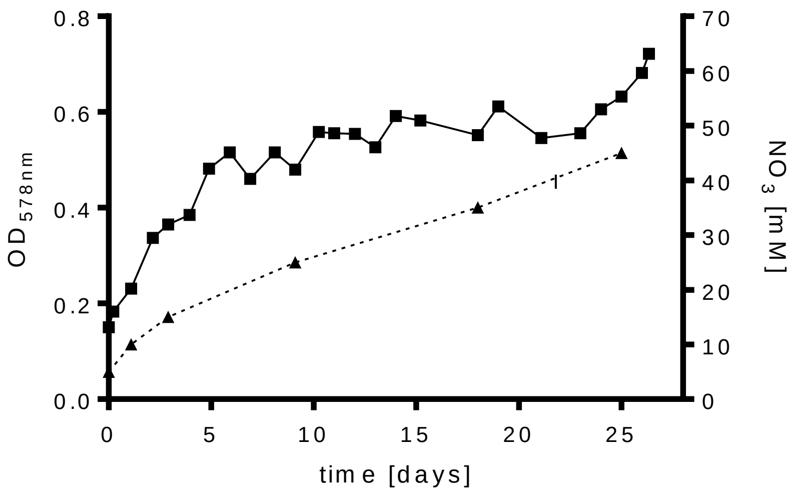

Fig. S4 Growth of *Stl. denitrificans* in a 200-L-fermenter with 3 mM β-sitosterol under denitrifying conditions. Cultivation was performed under nitrate-limitation (fed-batch culture); ■ increase of OD_{578nm}, ▲ increase in nitrate consumption.
