# Supplementary material for: Four Molybdenum-Dependent Steroid C-25 Hydroxylases: Heterologous Overproduction, Role in Steroid Degradation, and Application for 25-Hydroxyvitamin D3 Synthesis
Source: mBio. 2018 Jun 19;9(3):e00694-18. doi: 10.1128/mBio.00694-18 (PMC6016249; doi:10.1128/mBio.00694-18)
Supplement: TABLE S2 [file mbo003183935st2.docx]

**Table S2** Mass spectrometric analysis of enriched protein from *Stl. denitrificans* wild-type catalyzing β-sitost-4-en-3-one C25-hydroxylation. The excised bands correspond to those highlighted by an arrow in Fig. 5. The gene products identified correspond to α_4_β_4_γ_4_ genes of S25DH_4_ heterologously produced in *T. aromatica*.

| **Mass of excised SDS protein band** | **Gene product identified** | **Score** | **Sequence coverage** | **Mass deduced from amino acid sequence** |
| --- | --- | --- | --- | --- |
| 110 | SDENCHOL_20206 (α_4_) | 4187 | 62% | 108 |
| 55  50  37 | SDENCHOL_20206 (α_4_)  SDENCHOL_20206 (α_4_)  SDENCHOL_20205 (β_4_) | 2844  2561  1635 | 47%  42%  66% | 108  108  38 |
| 27 | SDENCHOL_20204 (γ_4_) | 983 | 47% | 23 |
