# Supplementary material for: Four Molybdenum-Dependent Steroid C-25 Hydroxylases: Heterologous Overproduction, Role in Steroid Degradation, and Application for 25-Hydroxyvitamin D3 Synthesis
Source: mBio. 2018 Jun 19;9(3):e00694-18. doi: 10.1128/mBio.00694-18 (PMC6016249; doi:10.1128/mBio.00694-18)
Supplement: TABLE S3 [file mbo003183935st3.docx]

**Table S3** Oligonucleotide primers used for heterologous production of steroid C25 dehydrogenases.

| **Primer** | **Sequence (5´-3´)** | **T_a_ (°C)** | **Restriction site** | |
| --- | --- | --- | --- | --- |
| S25DHα_1__for | cccaagcttttaactttataaggaggtgaagcatgcagatttccagacgt | 65 | | HindIII |
| S25DHα_1__rev | ccactagtcatcacctgaacattacgccgcccggctc | 65 | | SpeI |
| S25DHγ_3_β_3__for | GGTATCGATTGACCTAAGGAGGTAAATAATGAAAGTCACCTACACATC | 65 | | ClaI |
| S25DHγ_3_β_3__rev | GGTAAGCTTTCAAGCCCAGCTCAACGAATG | 62 | | HindIII |
| SdhD_for | cggactagtttaactttataaggaggtgatacatgcaaatgagcaatg | 66 | | SpeI |
| SdhD_rev | cgtctagacaaaaaccggaccgatgagcgatcaat | 66 | | XbaI |
| S25DHα_2__for | GCATCGACTAGTCACTATTTCGCCTTCTCGACCTCCAC | 61 | | HindIII |
| S25DHα_2__rev | GATTGAAAGCTTTTTAACTTTATAAGGAGGTGAAGCATGCAATTCATGCAATTGACCCG | 60 | | SpeI |
| S25DHα_3__for | GATGCAACTAGTCATCAGGCGCTGGCCTTTTCC | 63 | | HindIII |
| S25DHα_3__rev | GATCGTAAGCTTTTAACTTTATAAGGAGGTGAAGCATGCAAGTGTCACGTCGTCAC | 62 | | SpeI |
| Gibson_S25DHα_4_β_4_γ_4_SdhD_for | GGAAACAGAATTCGATACCGGGAGTACCAGTGAAAGTCACCTAC | - | | - |
| Gibson_S25DHα_4_β_4_γ_4_SdhD_rev | GCTGCAGGAATTCGATATCATTCAGACCAGCTTTCAGACCG | - | | - |
